# Impact of water flow rate on finishing pig performance

**DOI:** 10.1093/tas/txac125

**Published:** 2022-09-01

**Authors:** Hannah E Miller, Katlyn McClellan, Jorge Y Perez-Palencia, Ryan S Samuel, Crystal L Levesque, Robert C Thaler

**Affiliations:** Department of Animal Science, South Dakota State University, Brookings, SD 57007, USA; Department of Animal Sciences and Agricultural Education, Jordan College of Agriculture Sciences and Technology, Fresno State University, Fresno, CA 93740, USA; Department of Animal Science, South Dakota State University, Brookings, SD 57007, USA; Department of Animal Science, South Dakota State University, Brookings, SD 57007, USA; Department of Animal Science, South Dakota State University, Brookings, SD 57007, USA; Department of Animal Science, South Dakota State University, Brookings, SD 57007, USA

**Keywords:** finishing pigs, summer, water flow rate

## Abstract

A survey of 23 South Dakota pork producers in 2019 reported that 68% of the waterers in finishing barns had water flow rates above the recommended rate of 500–1,000 mL/min. The objective of the two studies was to determine the impact of water flow rate on finishing pig performance in the summer months. Study 1 used a total of 396 pigs in two groups in a 77-day trial (35.0 to 104.3 kg BW) with 6 pigs/pen and 1 cup waterer/pen. Study 2, conducted in a commercial style barn, used a total of 1,227 pigs in an 84-day trial (60.9 to 117.4 kg BW) with 26 pigs/pen and 2 cup waters/pen. Pens were assigned to one of three water flow rates (high, medium, low) based on the 3-hole settings of the water nipples (2.0, 1.0, and 0.8 mm; *n* = 22 and 16 pens/treatment for Study 1 and 2, respectively). Room temperature, outside temperature and relative humidity were recorded daily for both studies. In Study 1, water disappearance was recorded daily, and individual pen water flow rates were recorded every two weeks. At every diet phase change (26 ± 2.6 days), feed disappearance and individual pig body weights were recorded. Water flow rates averaged 1856 ± 188, 906 ± 214, 508 ± 100 mL/min for high, medium, and low flow settings, respectively. In Study 2, individual pen water flow rate, water disappearance, BW, and feed disappearance were recorded every two weeks. Water flow rates averaged 1115 ± 98, 906 ± 209, and 605 ± 203 mL/min for high, medium, and low flow settings, respectively. In both studies, there were no differences in final BW, cumulative ADG, or G:F. Due to the variability of water flow rate within a setting, data was further analyzed using regression with flow rate as the independent variable. Apart from average daily water disappearance (adj. *R*^2 ^= 0.87), there was a low relationship between pig performance and water flow rate (adj. *R*^2 ^< 0.09). The low *R*^2^ values associated with pig performance and the high association with water disappearance suggests that water flow rate above current recommendations has little impact on finishing pig performance but does contribute to water wastage and its associated costs.

## INTRODUCTION

Water is a vital part of all livestock production and is an important component for pig performance, cost of production, and environmental impact (Muhlbauer et al., 2010; Gerbens-Leenes et al., 2013). A recommended water flow rate has been outlined by the National Swine Nutrition Guide which states that water delivery for finishing pigs should be between 500 ando 1,000 mL/min (Brumm, 2010a). However, more recent work has shown that growing-finishing pigs can perform well on delivery rates as low as 250 mL/min (Brumm, 2010b). The same report (Brumm, 2010b) describes water flow rates of 1,000 mL/minute as “more than adequate”. A 2019 survey of South Dakota Pork Producers reported that 68% of the waterers tested had water flow rates above the recommend 1,000 mL/min water flow rate (Zeamer et al., 2021).

There is little evidence supporting that excess water consistently translates to improved pig performance. Pigs fed liquid diets with water to meal ratios ranging from 2:1 to 3.5:1 with supplementary water, responded with an increase in daily gain as water content in the meal increased, but no changes in feed intake were observed (Gill et al., 1986). Brooks et al. (1989) reported that increasing delivery rate (300–900 mL/min) increased water disappearance by 80% but it had no impact on daily gain or feed intake. Li et al. (2005) demonstrated in an experiment with growing-finishing pigs that neither drinker height nor water flow rate had an impact on feed intake or daily gain.

Water wasted from the water drinker accounts for 25–40% of the total water used in swine facilities (Li et al., 2005). Water nipples with higher flow rates tend to have increased water wastage and water spillage; for example, waterers set to 2,000 mL/min in a grow-finish trial had over 2 times the amount of water spillage compared to waterers within the recommended flow rate (Li et al., 2005). Muhlbauer et al. (2010) reported that pigs given free access to water consumed similar amounts of water, regardless of other factors including drinker height and design. Differences in water disappearance can most likely be attributed to wastage, rather than consumption (Muhlbauer et al., 2010). When comparing average water usage of growing-finishing pigs from farms in Canada, United States and Netherlands, the data show dramatic differences in water usage between the three countries (7.0, 17.0 and 4.6 L/pig/day, respectively) (DGH Engineering Ltd., 1999; North Carolina Cooperative Extension Service, 1999; Prairie Swine Centre, 2000; Froese and Small, 2001). The lower water usage of the Dutch pigs indicates that proper conservation techniques can reduce water usage without negatively impacting pig performance. Alternatively, excess water disappearance increases production costs associated with manure handling and storage costs (Mroz et al., 1995; Fleming et al., 1998; Muhlbauer et al., 2010).

The actual water flow rate of a drinker is a combination of external supply and internal variables. The water source may have limited ability to alter external supply; however, adjustments to the water flow rate can be made from different internal control points, including water pressure in the line as well as adjustments of valves and pumps and at the water nipple. We hypothesize that increasing the water flow rate beyond the recommendation of 1,000 mL/min will not impact finishing pig performance. Therefore, due to the potential impact on pig growth performance, cost of production, and sustainability, this study was conducted to evaluate the impact of water flow rate on finishing pig performance.

## MATERIALS AND METHODS

### Study 1

The experimental protocol used in this study was approved by the South Dakota State University Institutional Animal Care and Use Committee (IACUC 2006-028E) and followed the Guide for the Care and Use of Agricultural Animals in Research and Teaching (FASS, 2010). The experiment was performed in the wean-to-finish barn at South Dakota State University Swine Education and Research Facility, located in Brookings, SD 57006, USA.

#### Animals and housing

**Figure 1.**
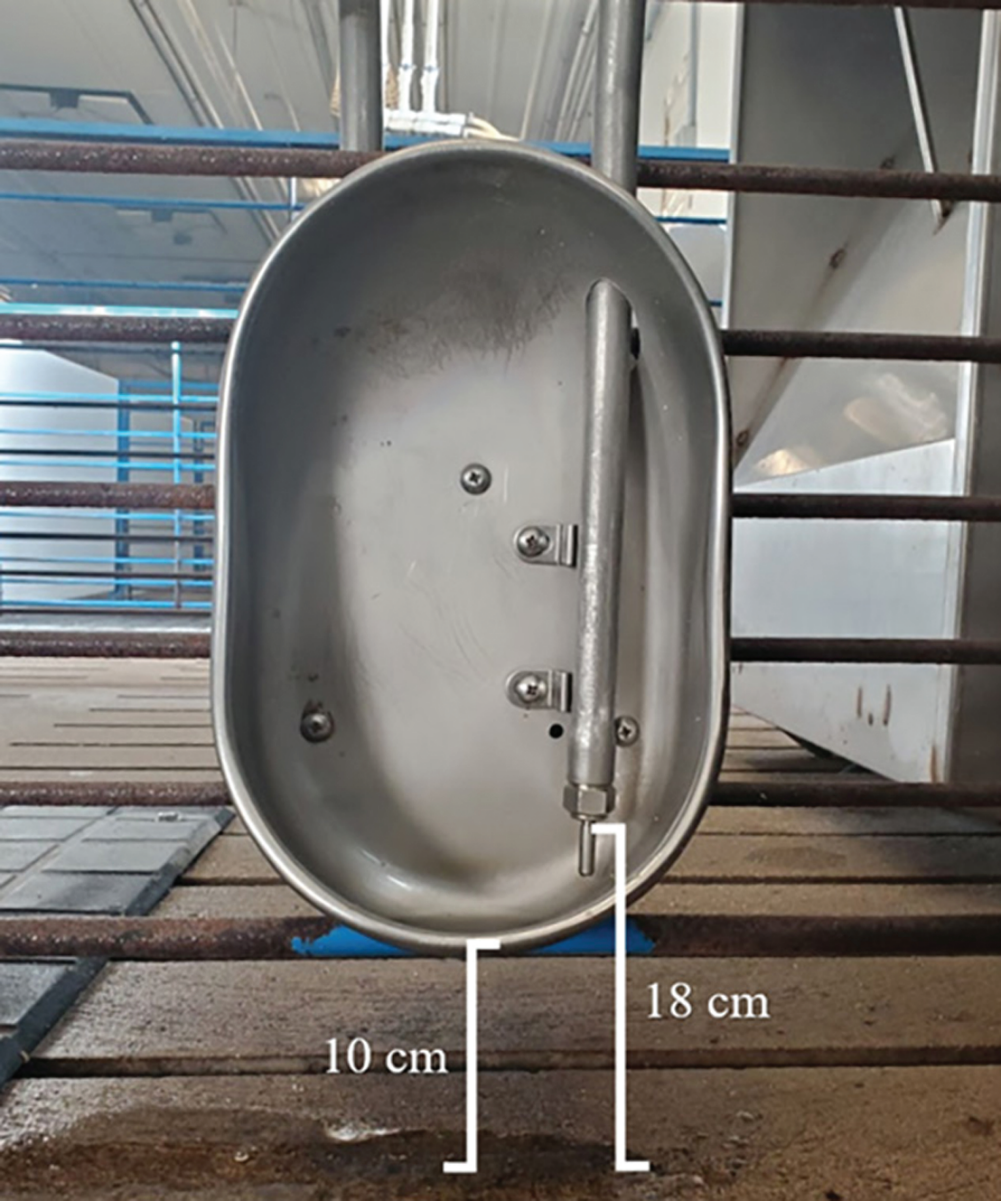
Dimensions of the water nipple and water cup.

A total of 396 barrows and gilts (Duroc; x PIC Camborough) (35 kg ± 4.6; 6 pigs/pen; 0.66 m^2^ per pig) in two groups in separate rooms, were utilized in a 77 ± 3.4-day trial until pigs reached a final weight of 104.3 kg. Pens were not remixed at the initation of the trial to balance for sex, as mixing pens can negativly impact performance due to fighting and establishment of hierarchy (Li and Wang, 2011). Pens were allocated to three different water setting groups, with a total of 22 replicates pens per group. All pens contained one, 2-space dry feeder and one cup waterer for ad libitum access to feed and water. The base of each water cup was approximately 10 cm above the slats (Fig. 1).

Data collection began June 2020 and concluded September 2020 for Group 1 and began July 2020 and concluded October 2020 for Group 2. The barn was mechanically ventilated; at the beginning of each group, the temperature setpoint was 19.4 °C and then decreased 0.06 °C daily until reaching 14.4 °C. High, low, and average room temperatures were recorded between 0600 and 0800 h daily. Outside temperature and relative humidity was collected through the Brookings Mesonet station (#BKMS2).

Throughout the trial, feed disappearance was monitored for each pen. All animals received common grow-finish diets in three phases, with 26 ± 2.6 days/phase (Table 1). Feed disappearance and body weight (BW) were measured at the end of each diet phase to determine average daily gain (ADG), daily feed intake (ADFI), and gain-to-feed ratio (G:F).

**Table 1.** Composition of Study 1 grow-finisher diets.

Item	Body Weight, kg		
	35–55	55–80	80–105
Ingredients, %			
Corn	79.25	83.3	86.54
Soybean meal, 46.5%	17.68	13.83	10.77
l-Lysine HCl	0.40	0.38	0.36
l-Threonine	0.13	0.12	0.11
dl-Methionine	0.08	0.07	0.04
l-Tryptophan	0.01	0.01	0.01
Monocalcium phosphate	0.98	0.85	0.76
Limestone	0.97	0.94	0.91
Salt	0.30	0.30	0.30
Nursery Vitamin premix^1^	0.05	0.05	0.05
Trace Mineral premix^2^	0.15	0.15	0.15
Total	100.0	100.0	100.0
Calculated analysis			
ME, kcal/kg	3333	3341	3346
NE, kcal/kg	2469	2500	2522
CP, %	15.3	13.9	12.7
Ca, %	0.62	0.57	0.54
P, %	0.27	0.24	0.22
Available P, %	0.27	0.23	0.21
SID^3^ amino acids, %			
Lys	0.94	0.84	0.75
Ile:Lys	0.55	0.55	0.55
Leu:Lys	1.36	1.42	1.51
Met:Lys	0.33	0.33	0.32
Thr:Lys	0.63	0.63	0.65
Trp:Lys	0.16	0.15	0.16
Val:Lys	0.65	0.65	0.67

J & R Distributing Inc. 518 Main Ave, Lake Norden, SD 57248—USA. Minimum provided per kg of diet: Calcium 55 mg, Vitamin A 11,000 IU, Vitamin D3 1,650 IU, Vitamin E 55 IU; Vitamin B12 0.044 mg, Menadione 4.4 mg, Biotin 0.165 mg, Folic Acid 1.1 mg, Niacin 55 mg, d-Pantothenic Acid 60.5 mg, Vitamin B16 3.3 mg, Riboflavin mg, 9.9 Thiamine 3.3 mg.

J & R Distributing Inc. 518 Main Ave, Lake Norden, SD 57248—USA. Minimum provided per kg of diet: Copper 16.5 ppm, Manganese 44.1 ppm, Selenium 0.03 ppm, Zinc 165 ppm.

SID = Standard ileal digestible.

Daily animal monitoring included observations of individual pigs, room environment, and facility conditions, as well as records of veterinary treatment on a per pen basis including number of pigs treated/pen, drug administered, dosage, duration, reason for pig removal (i.e., dead, untreatable health issue such as umbilical prolapse, morbidity), and evidence of health concerns (i.e., lameness, coughing).

#### Water Settings

**Figure 2.**
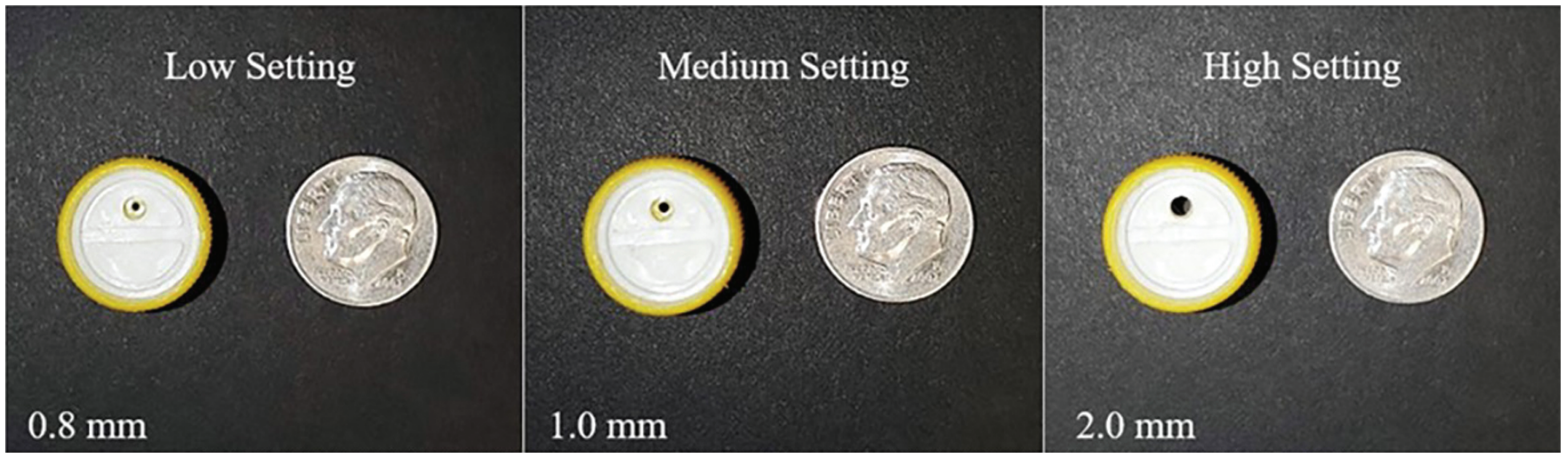
Size comparison of water nipple setting orifice diameter.

The three water settings were defined as low, medium, and high based on the three-hole diameters (0.8, 1.0, and 2.0 mm respectively; Fig. 2) of the commercial water nipples (Koca USA Inc., Des Moines, IA 50313) used in the facility. Recommended water flow rate for grow-finish pigs is between 500 and 1,000 mL/min (Brumm, 2010). For purpose of the study, tehe medium setting was considered within the range of the recommended water flow rate. Low and high settings were considered to be outside of the recommended water flow rate. Water flow rate of the cup waterer in each pen was recorded every two weeks by the same technician. Water flow rate was measured by letting the water overflow the water cups into a basin below and measuring the volume of water collected in 30 s, and then adjusted to one-minute flow rates.

Each room was equipped with 4 water lines, and each line fitted with an individual water meter (Dwyer Instruments Inc., Michigan City, IN 46361). Water for all pens on a given water nipple setting was supplied by a designated water line. From the meter, total water disappearance for each of the 3 settings was recorded daily.

### Study 2

The South Dakota State University Institutional Animal Care and Use committee approved the protocol (IACUC 2106-037E) used in this study. The experiment was conducted at the South Dakota State University commercial wean to finish research facility, located in Flandreau, SD 57028, USA.

#### Animal housing

In the second study, 1,227 barrow and gilts (PIC 800 × FAST/PIC) (60.9 kg ± 4.4; 26 pigs/pen; 0.82 m^2^ per pig) in were utilized in an 84-day trial, with a final body weight (117.4 kg) recorded on day 91, one day prior to the first marketing event. Pens were balanced for sex, with 13 barrows and 13 gilts per pen. Pens within a block were randomly assigned to one of the three different water settings, with 16 pens per setting. Pen dimensions were 3.1 m × 6.9 m and each pen contained one 5-slot stainless steel dry feeder and two cup waters placed 1 m apart on the same side of the pen and within 1 m of the feeder. The same model of drinker was used as in Study 1 (Fig. 1). Daily animal observations followed same protocol as Study 1.

Data collection began June 2021 continued through September 2021. The barn was mechanically ventilated with temperature setpoints at 20.5, 18.3, 16.7, and 16.1C on day 0, 27, 55, and 77, respectively. High, low, and average room temperature was recorded between 0600 and 0800 daily. Outside temperature and relative humidity was recorded through the Flandreau Mesonet Station (#FLNS2).

All pens received a common grow-finish diet (Table 2). Pen feed disappearance and BW were measured every 2 weeks for calculation of ADG, ADFI, and G:F.

**Table 2.** Composition of grow-finisher diets (Study 2).

Item	Body Weight, kg			
	25–55	55–75	75–90	90-marketing
Ingredients,%				
Corn	74.85	78.53	80.75	85.44
Soybean meal	12.65	9.15	9.63	7.50
DDGS	10.00	10.00	7.50	5.00
Limestone	0.925	0.87	0.82	0.80
Monocalcium Phosphate	0.15	0.07	0.05	0.11
Salt	0.50	0.50	0.5	0.50
Lysine-HCl	0.49	0.47	0.40	0.35
Threonine	0.165	0.15	0.12	0.10
dl-Methionine	0.05	0.03	-	-
Vitamin and Mineral Premix^1^	0.15	0.15	0.15	0.15
l-Tryptophan	0.04	0.04	0.03	0.025
Copper Chloride	0.025	0.025	0.025	0.025
Total	100.00	100.00	100.00	100.00
Calculated analysis				
ME, kcal/kg	2497	3303	3310	3317
NE, kcal/kg	3296	2518	2533	2559
CP, %	14.95	13.57	13.25	11.88
Ca, %	0.45	0.40	0.38	0.38
P, %	0.39	.37	0.34	0.34
Available P, %	0.12	0.12	0.13	0.12
SID^2^ amino acids, %				
Lys	0.90	0.80	.75	0.65
Ile:Lys	1.14	1.16	1.15	1.15
Leu:Lys	1.51	1.60	1.67	1.77
Met:Lys	0.77	0.80	0.79	0.81
Thr:Lys	1.10	1.12	1.12	1.13
Trp:Lys	0.27	0.27	0.27	0.28
Val:Lys	1.57	1.61	1.60	1.61

Provided per kilogram of the diet: 1,998 FTU phytase, 3,522 IU vitamin A, 1,101 IU vitamin D3, 22 IU vitamin E, 3.0 mg vitamin K3, 26.4 mg niacin, 17.6 mg pantothenic acid, 5.2 mg riboflavin, 23.8 ug vitamin B12, 30 mg Mn from manganous oxide, 100 mg Zn from zinc hydroxychloride, 80 mg Fe from ferrous sulfate, 12 mg Cu from copper chloride, 0.40 mg I from ethylenediamine dihydroiodide, and 0.30 mg Se from sodium selenite.

SID = Standard ileal digestible.

#### Water settings

Water flow rate was recorded every 2 weeks in the same manner as study 1. Each pen was equipped with an individual water meter and pen water disappearance was recorded every 2 weeks. Average daily water disappearance was calculated for each period in the study.

Total water usage during the 84-d experimental period per water setting was recorded, and water disappearance on a per pig basis was calculated. Information regarding water cost for livestock use was sourced from Mid Dakota Rural Water System (mdrws.com/billing/waterrates/).

### Statistical analysis

The UNIVARIATE procedure of SAS (SAS Inst., Inc., Cary, NC) was used to confirm the homogeneity of variance and to analyze for outliers. Data was analyzed using the PROC MIXED procedure in SAS. In the model, water flow was considered the main effect and pen the experimental unit. For study 1, room was the blocking factor. A ratio of gilts:barrows was included as a random effect to account for potential differences in performance between gilts and barrows. Location in the room was the blocking factor in Study 2. For both studies, Tukey’s adjusted means test was used to detect differences between water settings; data reported as least squares means ± SEM. Results were considered significant at *P *≤ 0.05 and a tendency at 0.05 < *P *≤ 0.1 for all statistical tests.

For Study 1, average daily barn water consumption per kilogram of BW and high barn temperature was analyzed using a regression model in SAS with room temperature as the independent variable. For both studies, water flow rate and pig performance were analyzed using regression model in SAS, with water flow rate as the independent variable. All regression models were tested under linear, quadratic, and cubic responses to corresponding variables. Responses were considered significant at *P *≤ 0.05.

## RESULTS

### Study 1

**Figure 3.**
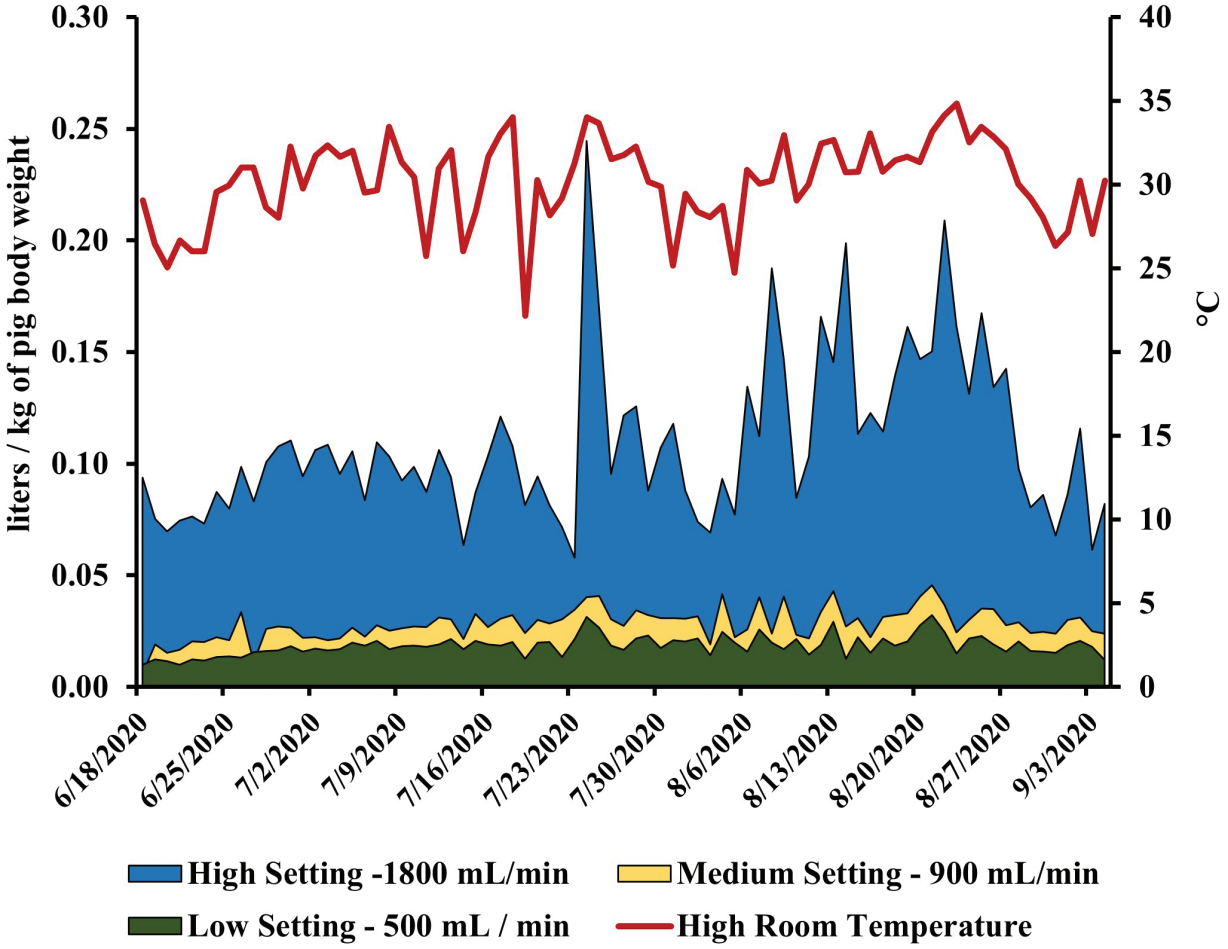
Average daily water disappearance per kg BW and high room temperature over time in Study 1.

**Figure 4.**
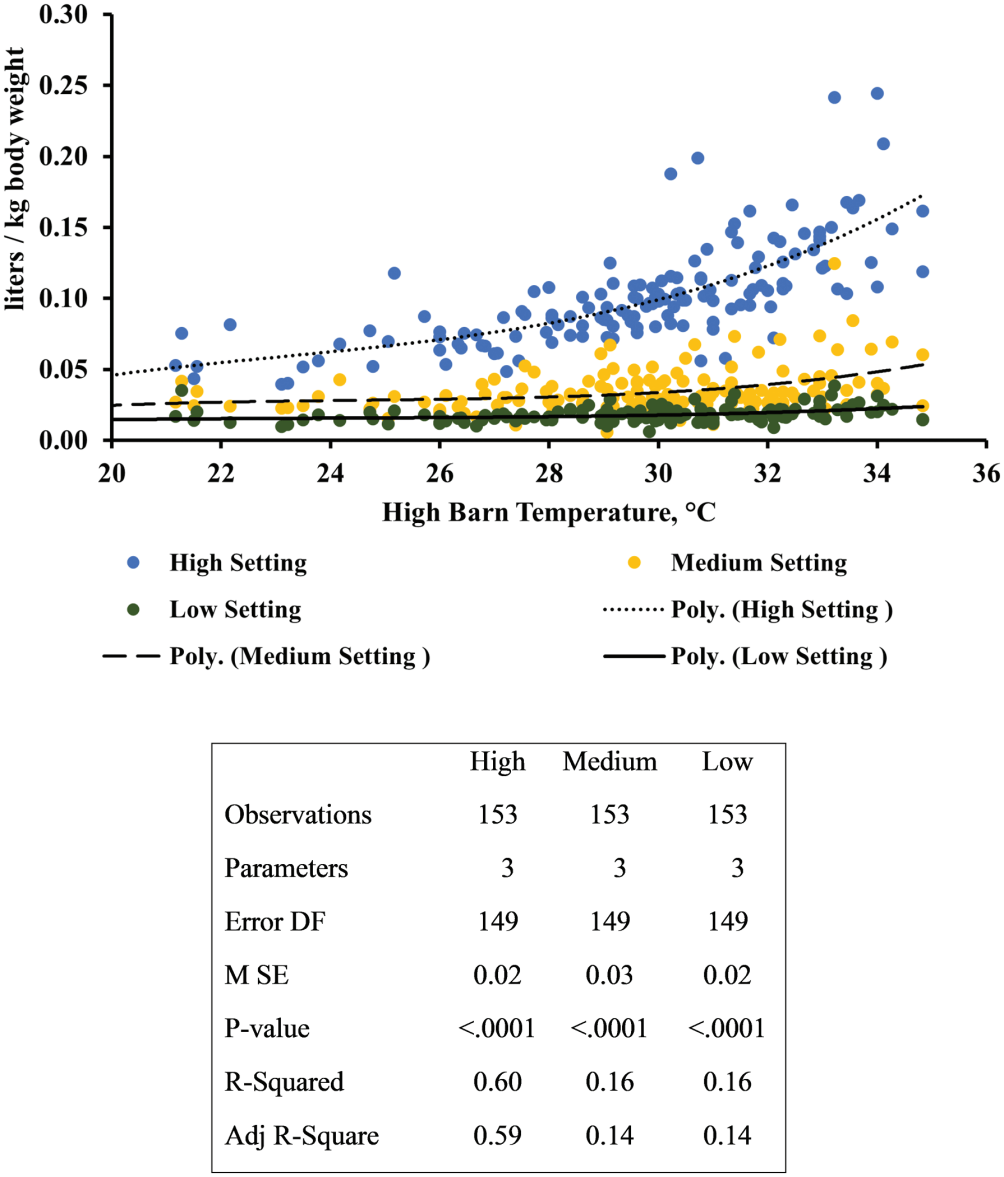
Regression of water usage per pig on the high, medium, and low water settings against daily high room temperature in Study 1.

Over the entire experimental period of Study 1, average daily barn temperature was 22.8 °C (*SD *= 3.3 °C) with daily average high temperature of 29.0 °C (*SD *= 3.7 °C), and daily average low temperature of 20.5 °C (*SD* = 2.9 °C). The average outside temperature and relative humidity, for the entire experiment period was 20.0 °C (*SD* = 4.9 °C) and 73.1% (*SD* = 7.9%), respectively. Water flow rates were 508 ± 100, 906 ± 214, and 1856 ± 188 mL/min for low, medium, and high settings, respectively. Daily water disappearance for the high, medium, and low settings were 6.8 ± 3.6, 2.3 ± 1.1, 1.3 ± 0.8 L/pig/d, respectively. Average daily water disappearance per kg BW and daily high room temperature (Fig. 3) demonstrates the pattern of increase and decrease in daily water consumption per water flow setting. The pattern of increased water disappearance with increased room temperature was especially prevalent for the high water setting with the medium and low setting maintaining a much more consistent daily water disappearance. A cubic regression (*P *< 0.0001) described the relationship between high barn temperature and water disappearance per kg of BW for each of the separate water settings (Fig. 4). Adjusted *R*^2^ values of each cubic regression for high, medium, and low water settings are 0.60, 0.14, and 0.14, respectively.

The ADG was greater (*P* = 0.04) for pens on the high water flow setting compared to low setting with the medium setting intermediate in Period 1 (Table 3). During this period, ADFI was greater for pens on high setting (*P *= 0.05) compared to pens on the low water setting. There were no differences in G:F during period 1. In Period 2, there were no differences in BW and ADG. However, ADFI was greater (*P *= 0.04) in pens on the high water flow setting than for pens on the low water settings. This resulted in improvements in G:F ratio (*P* = 0.04) for pigs on the low water setting compared to pigs on the high water setting. In period 3, there were no differences in final BW, ADG, ADFI, or G:F. From d 0 to d 77, there was no difference in ADG or G:F. Conversely, cumulative ADFI (*P* = 0.02) was greater for pens on the high water compared to pens on the low setting, with the medium water setting intermediate (Table 3).

**Table 3.** Effect of water flow rate on Study 1 finishing pig performance^1^.

	Water flow rate setting				
	Low	Medium	High	SEM	*P*-value
Item					
Avg water flow rate (mL/min)	508	906	1856		
Standard deviation	100	214	188		
Initial BW, kg	35.11	34.99	35.04	0.80	0.994
Period 1, d 0–25					
BW, kg	54.96	55.37	56.29	1.05	0.614
ADG, kg/d	0.82^b^	0.85^ab^	0.87^a^	0.01	0.040
ADFI, kg/d	1.46^b^	1.51^ab^	1.59^a^	0.04	0.049
G:F	0.59	0.59	0.57	0.02	0.676
Period 2, d 25–53					
BW, kg	78.49	78.44	79.98	1.31	0.548
ADG, kg/d	0.85	0.84	0.86	0.01	0.528
ADFI, kg/d	2.21^b^	2.24^ab^	2.34^a^	0.04	0.011
G:F	0.38^a^	0.38^ab^	0.37^b^	0.004	0.037
Period 3, d 53–77					
BW d 77, kg	103.18	103.24	105.91	1.35	0.251
ADG, kg/d	1.00	1.01	1.05	0.02	0.120
ADFI, kg/d	2.81	2.79	2.89	0.05	0.206
G:F	0.36	0.36	0.36	0.01	0.897
Period 1-3, d 0–77					
ADG, kg/d	0.88	0.88	0.91	0.01	0.203
ADFI, kg/d	2.16^b^	2.18^ab^	2.27^a^	0.04	0.024
G:F	0.41	0.40	0.40	0.004	0.278

Pigs were assigned to one of three water settings with 22 pens per treatment and 6 pigs per pen.

Least square means in the same row with different superscript letters differ (*P* < 0.05).

**Figure 5.**
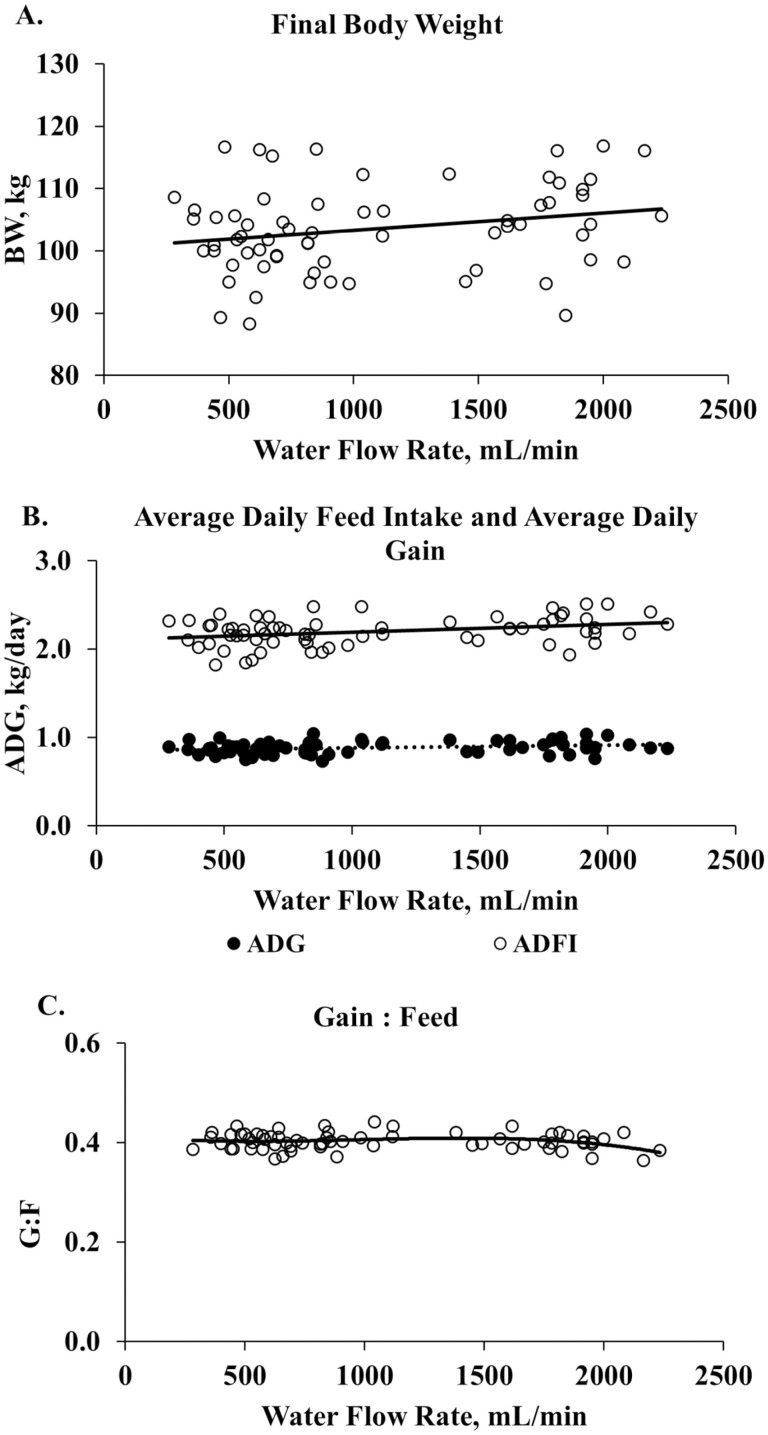
Regression of cumulative pig performance vs waterflow rate during Study 1. A = final body weight; B = cumulative average daily and feed intake; C = gain:feed ratio. Barn temperature ranged from 15.4 to 34.8 °C. High barn temperature averaged 29.0 °C for the entire trial period. Linear regression: body weight, *P* = 0.05, *R*^2^ = 0.04; average daily gain, *P* = 0.041, *R*^2^ = 0.05; average daily feed intake, *P* = 0.007, *R*^2^ = 0.09. Cubic regression: gain:feed ratio, *P* = 0.190.

Due to the variability in water flow rate within a setting, regression analysis was conducted to compare pen water flow rate and pig performance for each trial period. In Study 1 (Fig. 5), there was no relationship between water flow rate and final BW (Fig. 5A), cumulative ADG and ADFI (Fig. 5B), or G:F (Fig. 5C). Regression of performance within each weigh period are provided in Supplemental Figures S1 to S3.

### Study 2

In Study 2, barn temperature averaged 25.5 °C (*SD* = 2.3 c), with a daily average high temperature of 28.5 °C (*SD* = 2.8 °C) and daily average low temperature of 23.1 °C (*SD* = 5.8 °C). Outside average temperature was 21.4 °C (*SD =* 2.8 °C) and relative humidity was 73.9% (*SD* = 9.7%). Average water flow rates for the low, medium, and high setting were 605 ± 203, 906 ± 209, and 1115 ± 98 mL/min, respectively.

Differences in growth performance were only detected in Period 3 (Table 4) where there was a 2 kg reduction (*P* = 0.03) in BW between the low and the other two water settings with no difference in BW between pigs on the medium and high water setting (Table 4). Similarly, ADFI was lower for pigs on the low water setting relative to the other two settings (*P = *0.02). Both ADG and G:F were greater (*P* < 0.05) for pigs on the high setting compared to pigs on the low and medium settings.

**Table 4.** Effect of water flow rate on Study 2 finishing pig performance^1^.

	Water Flow Setting				
	Low	Medium	High	SEM	*P*-value
Item					
Avg water flow rate (mL/min)	605	906	1115		
Standard deviation	203	209	98		
Initial BW, kg	27.40	28.31	27.94	0.427	0.3568
Period 1, d 0 to 14					
BW, kg	36.70	37.54	37.45	0.497	0.4511
ADG, kg/d	0.65	0.66	0.68	0.010	0.2688
ADFI, kg/d	1.42	1.44	1.46	0.017	0.2208
G:F	0.46	0.46	0.46	0.005	0.6991
liters/ pig/day	0.10^c^	0.51^b^	0.79^a^	0.038	< 0.0001
Period 2, d 14–28					
BW, kg	51.37	53.00	52.34	0.592	0.1669
ADG, kg/d	1.10	1.08	1.08	0.015	0.769
ADFI, kg/d	1.78	1.81	1.82	0.015	0.1781
G:F	0.61	0.60	0.60	0.009	0.3375
liters/ pig/day	1.02^c^	3.10^b^	4.53^a^	0.220	< 0.0001
Period 3, d 28–42					
BW, kg	65.49^b^	67.54^a^	67.83^a^	0.629	0.0267
ADG, kg/d	1.01^b^	1.03^b^	1.11^a^	0.020	0.0040
ADFI, kg/d	2.24^b^	2.19^a^	2.26^a^	0.017	0.0212
G:F	0.46^b^	0.46^b^	0.49^a^	0.009	0.0318
liters/ pig/day	0.76^c^	2.83^b^	4.29^a^	0.280	< 0.0001
Period 4, d 42–56					
BW, kg	80.27	81.90	81.56	0.589	0.1366
ADG, kg/d	0.99	1.02	1.02	0.023	0.6702
ADFI, kg/d	2.43	2.45	2.47	0.021	0.4391
G:F	0.42	0.42	0.41	0.006	0.6860
liters/ pig/day	0.73^b^	4.50^a^	4.77^a^	0.306	< 0.0001
Period 5, d 56–70					
BW, kg	94.74	96.65	96.69	0.819	0.1756
ADG, kg/d	1.09	1.05	1.08	0.027	0.5963
ADFI, kg/d	2.71	2.71	2.71	0.027	0.9742
GF	0.40	0.39	0.40	0.008	0.4503
liters/ pig/day	0.30^c^	0.97^b^	1.29^a^	0.091	< 0.0001
Period 6, d 70–84					
BW, kg	109.61	111.68	111.78	0.963	0.2371
ADG, kg/d	1.12	1.07	1.08	0.018	0.2410
ADFI, kg/d	2.91	2.89	2.90	0.030	0.8040
G:F	0.38	0.37	0.37	0.004	0.2427
liters/ pig/day	1.05^c^	3.89^b^	5.31^a^	0.276	< 0.0001
Period 1-6, d 0–84					
ADG, kg/d	0.98	0.99	1.00	0.010	0.1444
ADFI, kg/d	2.24	2.25	2.27	0.015	0.3936
G:F	0.37	0.37	0.37	0.006	0.9934
liters/ pig/day	0.76^c^	3.32^b^	4.46^a^	0.216	< 0.0001
BW Day 91	115.81	118.14	118.08	1.001	0.1892

Pigs were assigned to one of three water settings with 22 pens per treatment and 16 pigs per pen.

Least square means in the same row with different superscript letters differ significantly (*P* < 0.05).

In all periods and for over the entire trial (Table 4), there were differences in average water disappearance on a per pig basis between all waterer settings (*P* < 0.0001), specifically in Periods 1, 2, 3, 5, and 6. In Period 4, the low setting had the lowest water disappearance, but there was no difference between the high and medium settings. From d 0 to 84, water disappearance (*P* < 0.0001) increased with the water nipple settings such that pigs on high water setting used 1.14 liters more per day than pigs on medium setting and 3.70 liters more per day than pigs on low setting. Over the 84-day experimental period pigs on the low, medium, and high settings used a total of 25,977, 114,080, and 151,350 L, respectively

**Figure 6.**
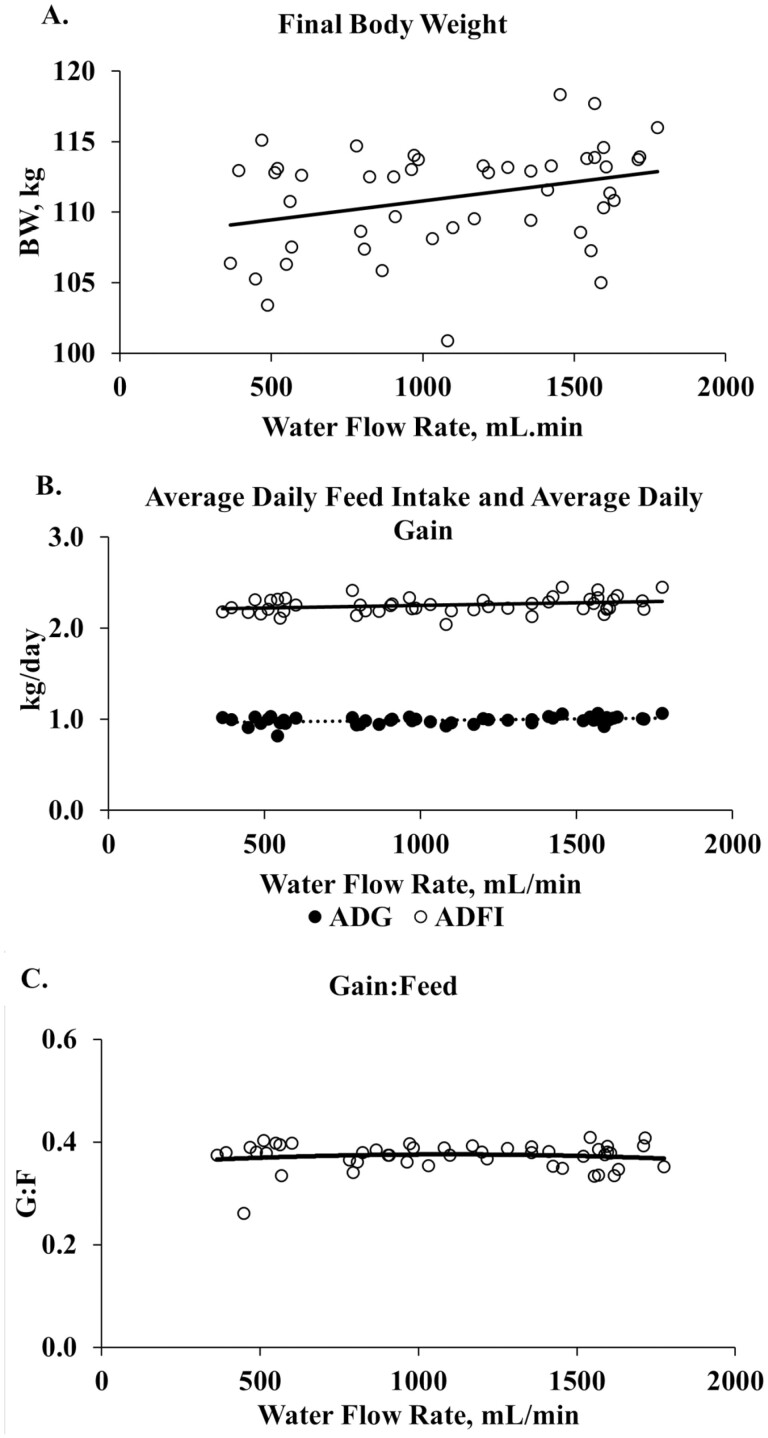
Regression of cumulative pig performance vs waterflow rate during Study 1. A = final body weight; B = cumulative average daily and feed intake; C = gain:feed ratio. Barn temperature ranged from 18.3 to 38.2 °C. High barn temperature averaged 28.5 °C for the entire trial period. Linear regression: body weight, *P* = 0.11; average daily gain, *P* = 0.01, *R*^2^ = 0.09; average daily feed intake, *P* = 0.05, *R*^2^ = 0.06. Cubic regression: gain:feed ratio, *P* = 0.79.

**Figure 7.**
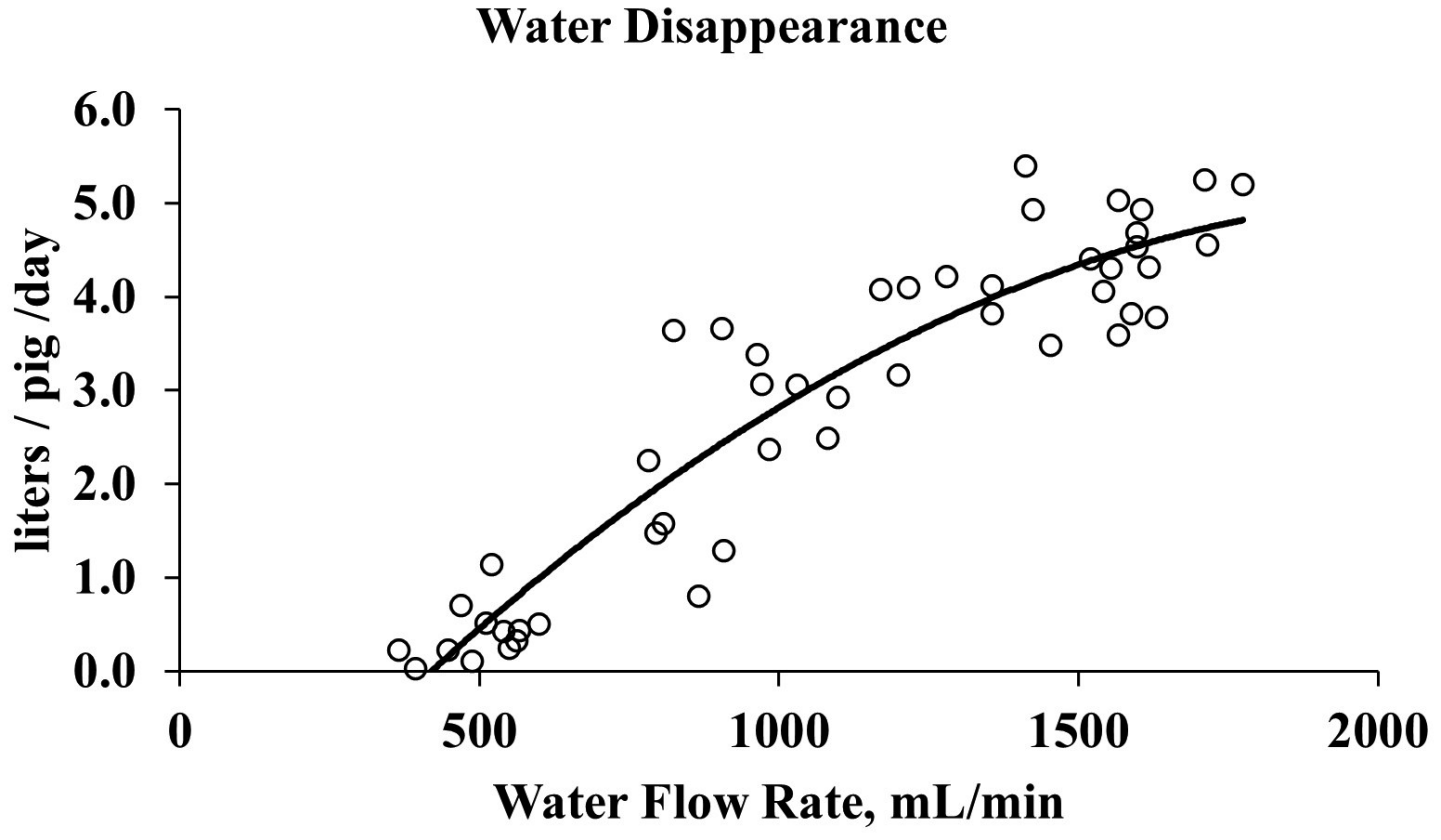
Regression of cumulative water disappearance in liters/pig/day. Quadratic regression, *P* < 0.0001, *R*^2^ = 0.87.

Similar to Study 1, regression of water flow rate against performance parameters were conducted. Regression of performance within each weigh period and d 91 BW are provided in Supplemental Figures S4–S10. In study 2 (Fig. 6) there was a linear increase in final BW (Fig. 6A), cumulative ADG, and ADFI (Fig. 6B) with increase water flow rate (*P* < 0.05; *R*^2 = ^0.08, 0.09, 0.06, respectively). There was no relationship between G:F (Fig. 6C) and water flow rate. While there was a positive quadratic relationship between water disappearance (Fig. 7) and water flow rate (*P* < 0.0001; *R*^2 ^= 0.87).

## DISCUSSION

The objective of these studies was to evaluate the impact of water flow rate on finishing pig performance. As referenced in the NRC (2012) water consumption “generally has a positive correlation with feed intake” and, hence, pig BW. A study from Li et al. (2005) reported no differences in water or feed intake due to water flow rate (between 500 and 1,000 mL/min) or nipple height. Additional water disappearance was attributed to water wastage. In the current studies, water disappearance increased as the water nipple setting increased. Although statistical differences in BW, ADG, ADFI, and G:F were noted in Period 3, Study 2, these differences were not maintained and resulted in no difference in cumulative pig performance. Results from Study 1 indicated a difference in cumulative ADFI between the high and low water settings, but similar to Li et al. (2005), this did not translate in an increase in final BW. From this it can be concluded that providing water above recommendation does not result in improved pig performance.

In the studies presented herein, variation in individual pen water flow rate within treatment may have reduced the ability to detect treatment differences. To address this, regression was used to evaluate the relationship between growth performance and pen water flow rate. The resulting low *R*^2^ values (i.e., <0.09) for all performance parameters, apart from water disappearance, supports the conclusion that water flow rate above current recommendation had little impact on pig performance.

It should be noted that Study 1was terminated at 104.3 kg BW and not at a market weight of 130 kg. It is possible that if pigs had been followed for an extended period of time, there could have been greater impact on performance at the heavier pig weights. However, given the lack of difference in gain in phase 3 of Study 1 and the resulting regression curve for ADFI and BW, it is unlikely that longer tracking of pig performance would have resulted in significant differences in growth. In addition, growth of pigs in Study 2 was followed through to market and the lack of difference provides further support to the conclusion that water flow rate above current recommendations does not improve pig performance. Similar to Li et al. (2005), the lack of improved pig performance in our trials suggest that the greater water disappearance on the high water setting could be considered wasted, rather than consumed by the pig, and thus added to the pit volume.

Producer manuals recommend finishing pigs receive 7 to 12 L of water per day (National Pork Board, 2018). Others, like Almond (1995) and Yang et al. (1981), recommend a higher level of water for finishing pigs (8–12 L/pig/day and 60 mL per kilogram of body weight, respectively). In these studies, even pigs on the high water setting utilized considerably less water than that of the recommendations from production manuals. In three experiments conducted by Li et al. (2005), the grow-finish pigs had lower water disappearances (1.94–7.31 L) than the above recommendations. This is most likely due to water requirements often being over-estimated and water wastage is not always considered (NRC, 2012). Due to the importance of water in many metabolic functions and the many variables that contribute to the level of intake, defining true requirements has proven challenging (NRC, 2012).

Water quality was not evaluated in the presented studies. For Study 1 and 2 water was sourced from Brookings Municipal Water Services and Big Sioux Community Water System, respectively. Both sources are of good quality and meet the standards for human consumption thus not expected to have influenced water usage in this study (Lozinski et al., 2022).

Throughout the duration of these studies, average barn temperature was well above thermal neutral zone for finishing pigs (Midwest Plan Service, 1982; Lammers et al., 2007). Higher ambient temperatures can lead to increased water consumption (Nienaber and Hahn, 1984; Li et al., 2005). Almond (1995) states that there is a greater than 50% increase in water consumption when temperatures increase from 12–15 °C to 30–32 °C. The same study found that during warmer temperatures, providing water at a higher flow rate may help to compensate for low ADFI typically associated with heat stress associated with the high ambient temperatures of the summer months (Almond, 1995). In a study utilizing pigs from 10 to 14 weeks of age, Nienaber and Hahn (1984) reported that as temperature and water flow rate increased, so did water consumption. Based upon observations from this study, usage from the high water setting appeared to follow a similar pattern of increasing as the room temperature increased, while pens on the medium or low water flow rate had lesser daily fluctuation in water disappearance, regardless of temperature. One possible explanation for this is that the pigs on the high water setting were using the water for other purposes besides consumption, for example, play or dispersing water to cool themselves on especially warm days (Meizhi et al., 2017; Chen et al., 2020).

High water usage is associated with increased water wastage (Nienaber and Hahn, 1984; Li et al., 2005). It is estimated that finishing pigs may waste up to 60% of the water used (Brooks, 1994). While water management and resources to conserve water (i.e., the introduction of the cup waterer) has improved over the years, water management still holds relevance to the industry today as demonstrated in a producer survey conducted by Zeamer et al. (2021), which shows the majority of swine producers providing water in excess. In Study 2, the regression curves and ANOVA table show how increasing pen water flow rate increases daily pen water disappearance. Increases in water usage has the potential to add additional costs for the producer without improving pig performance. Sourced from Mid Dakota Rural Water System (at mdrws.com/billing/waterrates on April 2022), a barn’s yearly water usage cost per liter is divided into 3 water usage categories, where fee per liter increases with greater water use (i.e., < 1.1 million L, 1.1–2.6 million L, and ≥ 2.6 million L equate to $1.06, $1.32, and $1.91 USD per 1,000 L, respectively). Using average water consumption data from Study 2, a single turn of 2400 head grow-finish barn, in a 125-day period, would use approximately 1.3 million, 996,000, or 228,000 liters of water, if the pigs had access to water settings equivalent to the high, medium, and low settings in this study. In this example, pigs on the high water setting would incur a 1.3 times greater water cost per pig ($1.06) relative to those on the medium water setting ($0.79), and a 10.6 times greater water cost per pig compared to those on the low water setting ($0.10).

Beyond the potential additional costs associated with greater water usage, pigs on the high water setting have the potential to incur more costs by adding excess water to the manure pit volume. Greutink (1993), Mroz et al. (1995), and Li et al. (2005) all found that as water disappearance increased, so did manure volume. Wasted water from drinkers and washing may increase manure volume by 10–30% (Chastain et al., 1998), resulting in a greater volume that needs to be removed from the pit and decreasing nutrient concentrations in the slurry. The nutrient content of a manure slurry combined with delivery and handling cost associated with the of manure is what determines its value (Fleming et al., 1998). Wasted water increases the water content of the manure slurry, which not only increases the quantity of slurry that must be transported but also creates a less nutrient dense product. This combination of increased handling cost and lower nutrient density, adds to the increase costs already associated with high water usage. While manure pit volume was not measured in either of the presented studies, further work is needed to confirm that increased water flow rate in finishing pigs results in increased manure pit volume.

Overall, water provided at a flow rate above current recommendation provides little benefit to pig performance. In both studies, there was no difference in cumulative feed conversion, ADG or final BW. Pigs on the high water setting did have a higher water disappearance than those on the medium and low water setting. It may be concluded that the additional water disappearance is attributed to play and wastage rather than being consumed by the pig. This ultimately adds to the manure pit volume which can lead to additional costs to the producer either through increasing the water bill and cost of manure handling. Due to costs of production and concerns related to increasing water wastage, swine producers are encouraged to frequently measure nipple flow rate and adjust when outside of accepted limits.

## Supplementary Material

txac125_suppl_Supplementary_Figures
