# Supplementary material for: Impact of water flow rate on finishing pig performance
Source: Transl Anim Sci. 2022 Sep 1;6(3):txac125. doi: 10.1093/tas/txac125 (PMC9512102; doi:10.1093/tas/txac125)
Supplement: txac125_suppl_Supplementary_Figures [file txac125_suppl_supplementary_figures.docx]

**B.**

**S1.**

**S2.**

**S3.**

**S4.**

**S5.**

**S6.**

**S7.**

**B.**

**D.**

**S8.**

**A.**

**D.**

**C.**

**S9.**

**S10.**

**S1**. Day 0 to Day 25 (Period 1), regression of pig performance vs waterflow rate during Study 1. A = body weight; B = average daily feed intake and gain; C = gain:feed. High barn temperature averaged 29.7 °C. Linear regression: body weight, *P* = 0.39; average daily gain, *P* = 0.44. Cubic regression: average daily feed intake, *P* = 0.28; gain:feed, *P* = 0.30.

**S2.** Day 25 to Day 53 (Period 2), regression of pig performance vs waterflow rate during Study 1. A = body weight; B = average daily gain and feed intake; C = gain:feed. . High barn temperature averaged 29.2 °C. Linear regression: body weight, *P* = 0.21; average daily gain *P* = 0.32; average daily gain, *P* = 0.005, R^2^ = 0.10; gain:feed, *P* = 0.02, R^2^ = 0.08.

**S3.** Day 53 to Day 77 (Period 3), regression of pig performance vs waterflow rate during Study 1. A = body weight; B = average daily gain and feed intake; C = gain:feed. High barn temperature averaged 27.9 °C. Linear regression: body weight, *P* = 0.05, R^2^ = 0.04; average daily gain, *P* = 0.05, R^2^ = 0.05; average daily feed intake, *P* = 0.64; gain:feed, *P* = 0.96.

**S4.** Day 0 to Day 14 (Period 1), regression of pig performance vs waterflow rate during Study 2. A = body weight; B = average daily gain and feed intake C = gain:feed; D = water disappearance. High barn temperature averaged 31.3°C. Linear regression: body weight, *P* = 0.34; average daily gain, *P* = 0.32; average daily feed intake, *P* = 0.03, R^2^ = 0.07, water disappearance, regression *P* <0.0001, R^2^ = 0.84. Quadratic regression: gain:feed, *P* = 0.55.

**S5.** Day 14 to Day 28 (Period 2) regression of pig performance (BW (a), ADG (b), ADFI (c), G:F (d), water disappearance (e) vs waterflow rate during Study 2. A = body weight; B = average daily gain and feed intake C = gain:feed; D = water disappearance. High barn temperature averaged 28.5°C. Linear regression: body weight, *P* = 0.12; average daily gain, *P* = 0.57; average daily feed intake, *P* = 0.05, R^2^ = 0.06; gain:feed, *P* = 0.10; water disappearance, *P* <0.0001, R^2^ = 0.81.

**S6.** Day 28 to Day 42 (Period 3) regression of pig performance vs waterflow rate during Study 2. A = body weight; B = average daily gain and feed intake; C = gain:feed; D = water disappearance. High barn temperature averaged 25.9°C. Linear regression: body weight, *P* = 0.008, R^2^ = 0.12; average daily feed intake, *P* = 0.003, R^2^ = 0.16. Cubic regression: average daily gain, *P* = 0.03, R^2^ = 0.27; gain:feed, *P* = 0.006, R^2^ = 0.19; water disappearance, *P* <0.0001, R^2^ = 0.81.

**S7.** Day 42 to Day 56 (Period 4) regression of pig performance vs waterflow rate during Study 2. A = body weight; B = average daily gain and feed intake C = gain:feed; D = water disappearance. High barn temperature averaged 28.2°C. Linear regression: body weight, *P* = 0.0041, R^2^ = 0.28; average daily feed intake, *P* = 0.14. Quadratic regression: average daily feed intake, *P* = 0.19; gain:feed, regression *P* = 0.23; water disappearance, *P* <0.0001, R^2^ = 0.61.

**S8.** Day 56 to Day 70 (Period 5) regression of pig performance vs waterflow rate during Study 2. A = body weight; B = average daily gain and feed intake; C = gain:feed; D = water disappearance. High barn temperature averaged 28.0°C. Linear regression: body weight, *P* = 0.04, R^2^ = 0.07. Cubic regression: average daily gain, *P* = 0.57; average daily feed intake, *P* = 0.34. Quadratic regression: gain:feed, *P* = 0.87; water disappearance, *P* <0.0001, R^2^ = 0.68.

**S9.** Day 70 to Day 84 (Period 6) regression of pig performance vs waterflow rate during Study 2. A = body weight; B = average daily gain and feed intake; C = gain:feed; D = water disappearance. High barn temperature averaged 27.7°C. Linear regression: body weight *P* = 0.03, R^2^ = 0.08; gain:feed *P* = 0.28. Quadratic regression: average daily gain, *P* = 0.23; average daily feed intake *P* = 0.20. Cubic regression: water disappearance, *P* <0.0001, R^2^ = 0.82.

**S10.** Regression of D 91 BW. Linear regression: *P* = 0.11
